# A Novel 3D Printed Multi‐Material Simulator for Endoscopic Stapes Surgery: The “3D Stapes Trainer”

**DOI:** 10.1002/lary.32168

**Published:** 2025-04-07

**Authors:** Giulia Molinari, Nicolas Emiliani, Laura Cercenelli, Barbara Bortolani, Rossana D'Azzeo, Arianna Burato, Livio Presutti, Gabriele Molteni, Emanuela Marcelli

**Affiliations:** ^1^ Department of Otolaryngology‐Head and Neck Surgery IRCCS Azienda Ospedaliero‐Universitaria of Bologna Bologna Italy; ^2^ Department of Medical and Surgical Sciences (DIMEC) University of Bologna Bologna Italy; ^3^ eDIMES Lab‐Laboratory of Bioengineering, Department of Medical and Surgical Sciences (DIMEC) University of Bologna Bologna Italy

**Keywords:** 3D printing, endoscopic ear surgery, simulation, stapes surgery, surgical training

## Abstract

**Objective:**

To develop and preliminarily validate a 3D‐printed, multi‐material, patient‐specific simulator of the external and middle ear affected by stapes fixation. The simulator was designed for training in endoscopic stapes surgery (SS), addressing the lack of reliable training platforms for this technically challenging procedure.

**Methods:**

Imaging data from a CT scan of a patient were used to create a virtual 3D model of significant ear structures. The simulator consisted of a multi‐use temporal bone holder and a single‐use middle ear box, printed with material Jetting 3D printing technology. Eight participants to a university ear surgery course used the simulator to perform endoscopic stapes surgery. The surgical performance was evaluated using modified Objective Structured Assessment of Technical Skills (OSATS) scoring, and participant feedback was gathered through qualitative questionnaires.

**Results:**

Seven of the eight participants successfully completed the simulated SS, with a mean surgical time of 24 min. OSATS scores showed acceptable performance, with 75% of participants achieving a score above 20. The tactile feedback, particularly for the stapes fixation, was well received, although the chorda tympani was deemed too fragile. The simulator was highly valued for visuomotor coordination development.

**Conclusions:**

The 3D Stapes Trainer represents a promising platform for training in endoscopic SS. Despite its limitations, the model provided young surgeons with a valuable platform to gain confidence in the steps of endoscopic SS, offering a high‐fidelity simulation that contributes to the development of the technical skills required in this demanding procedure.

**Level of Evidence:**

N/A

## Graphical abstract

The 3D Stapes Trainer represents a promising platform for training in endoscopic stapes surgery. This simulator consists of a multi‐use temporal bone holder and a single‐use middle ear box, printed with material Jetting 3D printing technology. Eight participants in a university ear surgery course used the simulator to perform endoscopic stapes surgery.

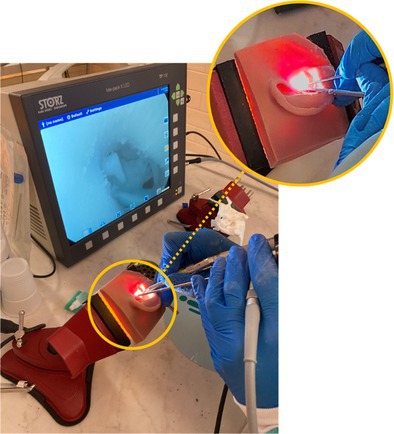

## 1 Introduction

Otosclerosis affects the otic capsule of the temporal bone (TB) and causes the ankylosis of the stapedo‐vestibular joint, resulting in progressive conductive or mixed hearing loss [1, 2].

The gold standard treatment for clinically significant otosclerosis (air‐bone gap ≥ 25 dB) is surgical, primarily performed with stapedotomy, a procedure where a fenestration on the footplate is created and a prosthesis is positioned to replace the fixed stapes [3, 4, 5, 6].

Stapes surgery (SS) could be performed through two possible approaches, microscopic and endoscopic, which are comparable in terms of audiological outcomes and complication rates [7, 8, 9]. However, the endoscopic approach has shown a superior educational value for surgeons in training, thanks to the better exposure of the middle ear (ME) structures and visualization of the surgical steps [7, 10, 11].

Despite being conceptually straightforward, SS is technically challenging and remains a highly functional surgery, performed on patients whose otosclerosis‐related quality of life is usually affected by hearing loss only. Comprehensive knowledge of the ME anatomy, combined with the development of manual dexterity and precision, accounts for the long learning curve of SS.

Currently, a dedicated reliable training platform for SS is lacking. Human cadaveric specimens are valid in terms of anatomical fidelity, but their availability is limited due to significant costs and regulatory constraints. The ex vivo ovine model is a feasible and effective training tool, even for advanced and salvage SS [12]. However, the main limitation of both these tools is the absence of pathology, implying that in both cases the footplate is not fixed and is easily prone to dislocation or fracture, preventing a realistic simulation of SS.

The restrictions of traditional training methods have led to the investigation of alternatives, among which synthetic models created with 3‐dimensional (3D) printing. They are perceived to be promising for TB surgical training, thanks to the possibility of creating patient‐specific pathologic simulators [13, 14]. So far, most of the 3D printed models focus on TB dissection [15, 16, 17, 18, 19, 20]; only a few of them allow endoscopic ear surgery, and none is specific for SS [21, 22, 23, 24, 25, 26, 27, 28, 29].

The aim of this study was to develop a 3D printed multi‐material simulator of the external and ME affected by stapes fixation. Its validity as a simulator for endoscopic SS was preliminarily investigated among a group of surgeons during an ear surgery course at the University Hospital of Bologna.

## 2 Materials and Methods

### 2.1 Image Segmentation and 3D Modeling

The Digital Imaging and Communications in Medicine (DICOM) data from a computed tomography (CT) scan with a slice thickness of 0.6 mm, from a 25‐year‐old patient with normal middle and external ear anatomy, was imported into Mimics 26.0 Medical software (Materialise; Leuven, Belgium). The patient gave informed consent for medical imaging to be used anonymously for scientific purposes. The study was approved by the Bioethics Committee of the University of Bologna (protocol n. 0289901, 09.10.2023).

Image segmentation was performed to create the virtual models of the ossicular chain (OC) (malleus, incus, stapes), the facial nerve, the posterior intraosseous segment of the chorda tympani (ChT), and the TB. Due to the imaging resolution, the intratympanic tract of the ChT [30], the stapedial tendon, the suspensory ligaments of the incus and malleus, and the incudo‐malleolar and incudo‐stapedial joints were added manually to the virtual 3D reconstruction under the supervision of an ear surgeon (G.M.).

### 2.2 Design of the Simulator and 3D Printing

The simulator was designed to consist of a single‐use removable part (ME box) and a multi‐use fixed part (TB holder).

**FIGURE 1:**
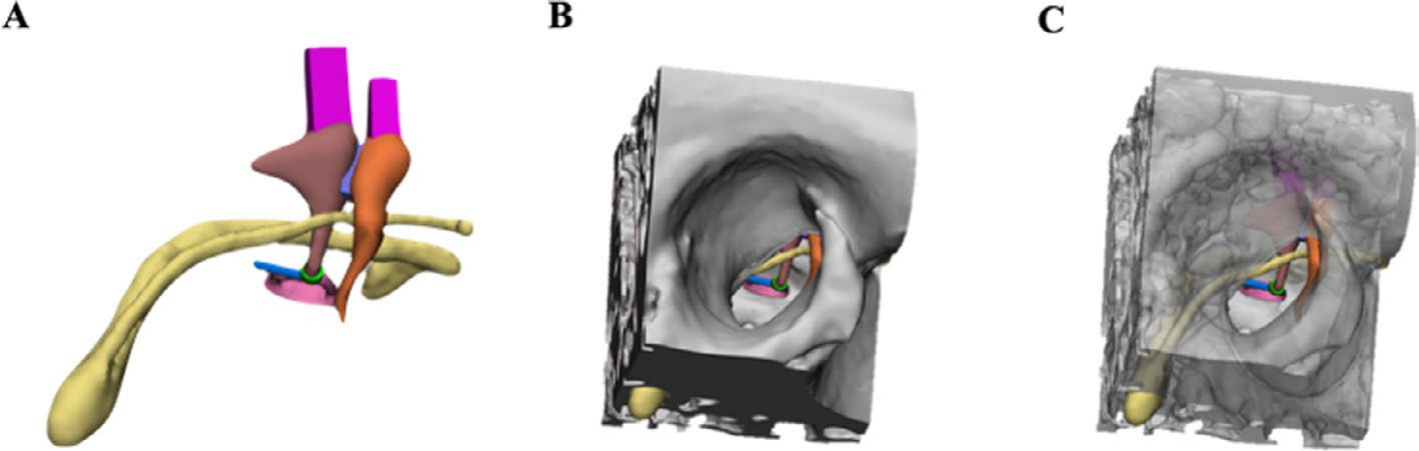
(A) View of the virtual model of the middle ear structures. Yellow: Facial nerve and chorda tympani; orange: Malleus; brown: Incus; pink: Stapes; magenta: Simplified suspensory ligaments of the incus and malleus; violet: Incudo‐malleolar joint; green: Incudo‐stapedial joint; blue: Stapedial tendon. (B, C) Middle ear structures visible inside the ME box through transparent and opaque bone, respectively.

**FIGURE 2:**
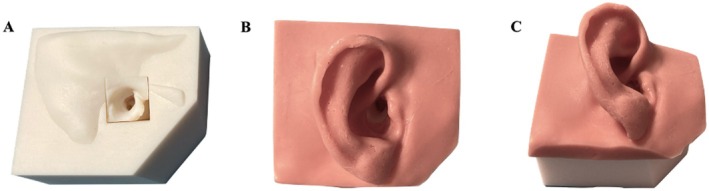
Final assembly of the simulator. (A) Middle ear (ME) box inserted into the temporal bone (TB) holder; anterior (B) and lateral (C) view of the external silicone ear placed on the assembled model.

The ME box was made by a rectangular cut of the TB centered on the external auditory canal, to include all the anatomical structures of the ME and the posterior canaliculus of the ChT (Figure 1). The TB holder fixed component was designed as a trapezoidal base, to simulate the surgical field, as well as to allow gripping of the simulator (Figure 2A). The external ear was replicated with silicone from the same CT scan using a specific mold, to serve as a support for the endoscope and instruments during the procedure (Figure 2B,C).

3D printing technology with stereolithography using Form 3B printer (Formlabs; Somerville, USA) was chosen for the TB holder (made with standard White V4 resin) and the external ear mold (printed with standard Clear V4 resin). The ME box was created using Material Jetting 3D printing technology, using the Stratasys J750 Digital Anatomy printer (Bio3DModel, SolidWorld; Firenze, Italy). This technology allows the fabrication of multi‐material parts, with the bony part printed in rigid material, whereas the soft tissues realized with a combination of rigid and rubber‐like materials to achieve realistic feedback. To replicate fixed stapes as in otosclerosis, the oval window margins (corresponding to the annular ligament) were printed with the same consistency as the surrounding bony structures. The printing time was about 1 h for each ME box and 5 h for the TB holder. The external ear was realized using Dragon Skin FX‐Pro bi‐component silicone and placed over the TB holder containing the ME box (Figure 2). After several endoscopic SS simulation sessions and progressive improvements of the model, the final version of the simulator was reached and called “3D Stapes Trainer” (3DST).

### 2.3 Stapes Surgery Simulation

The 3DST was tested during an educational event, the Bologna Oto Summer School (BOSS), a 5‐day combination of theoretical and practical sessions on ear surgery, with dissections on synthetic 3D printed models, ovine and cadaveric specimens, that took place at the University of Bologna—Alma Mater Studiorum, and at the IRCCS Azienda Ospedaliero‐Universitaria di Bologna (Italy), in June 2024.

The 8 participants to the BOSS attended a 20‐min lesson on SS, including an instructional video on endoscopic SS on the 3DST performed by an experienced surgeon, according to the standard stapedotomy technique by Fisch (see Video S1) [7]. Participants were allowed to watch the video as many times as needed.

**FIGURE 3:**
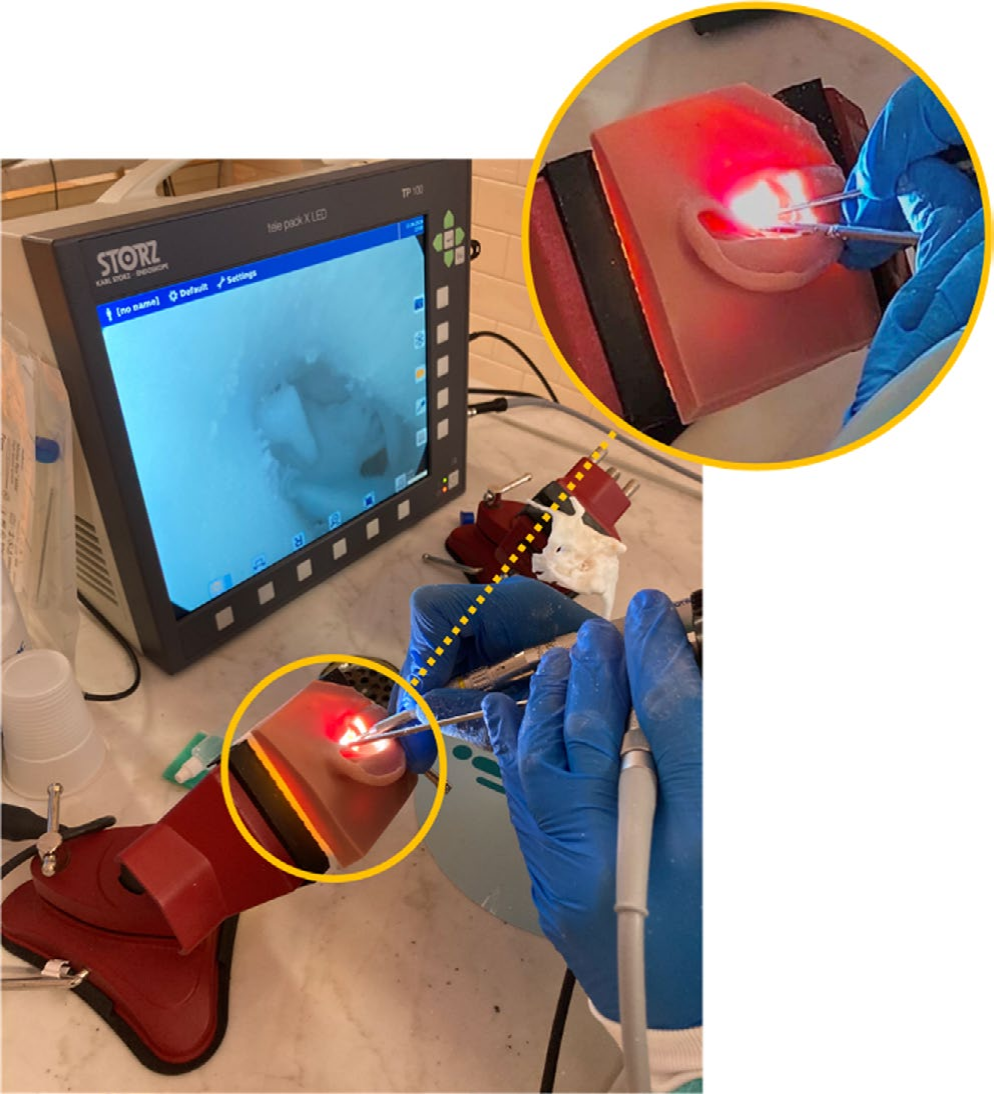
Set up of the endoscopic stapes surgery training on the 3D stapes trainer (3DST). The equipment consisted of an endoscopic telepack system with integrated light source connected to a high‐definition camera and a 3 mm 0° endoscope. An otological drill is held in the dominant hand to perform posterior crurotomy.

A standard instrument set for endoscopic SS was provided, consisting of a 3 mm diameter, 14 cm length, 0° rigid endoscope, connected to a high‐definition digital camera and xenon light source, a drill with a 0.6 diamond burr, a suction system, a House curette, a 1‐mm hook, Hartmann forceps, and crimping forceps (Figure 3).

All surgical simulations were recorded from the camera system and stored in an archive, for further evaluation by two of the authors (G.M., R.D.), who scored them, once anonymized, according to a modified versions of two previously validated scoring systems following the Objective Structured Assessment of Technical Skills (OSATS) format (Tables S1 and S2) [31]. The first was a global rating scale, whereas the second included specific items, according to the steps of SS, ranging from 1 to 5. Possible disagreements between the two raters were analyzed and a final score was chosen after thorough discussion. The item “measure length incus to footplate” was ignored because this step was not performed in the standard technique at our Institution. Intraoperative challenges and surgical errors were also annotated.

An anonymous questionnaire was administered to the participants at the end of the training sessions, investigating three aspects of the 3DST: anatomical fidelity and tactile feedback in comparison to the cadaver; reproducibility of the surgical steps; usefulness for training. A part for free comments was available.

## 3 Results

Of the 8 participants who took part in the training session, 5 were ENT specialists with less than 4 years of experience, among whom one (participant #3—P3) had experience in endoscopic SS, whereas two (participant #1 and #4) had experience in microscopic SS on patients. Participant #2 and #5 had no surgical experience in SS at all. The remaining three participants (participant #6, #7, #8) were ENT residents with no surgical experience with patients.

Seven out of 8 (87.5%) participants managed to complete the endoscopic SS. The mean surgical time to complete the task was 24 min (range 14.3–36.3 min). The operative time did not correlate with the overall OSATS score, with the fastest surgeon (P6) receiving the lowest OSATS scores.

### 3.1 General and Stapes‐Specific OSATS Scoring and Intraoperative Assessment

**FIGURE 4:**
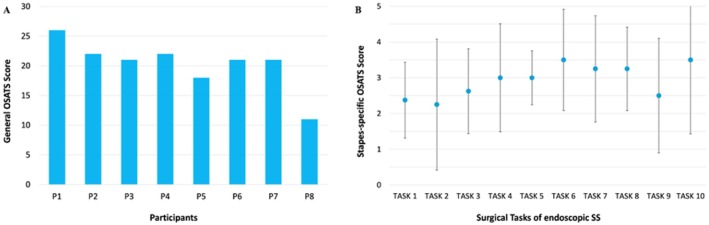
(A) Cumulative general OSATS scores from each participant (P1‐P8), over a maximum of 30. (B) The light blue sphere represents the mean OSATS score obtained from each task, while the bar represents the standard deviation. SS, stapes surgery.

Figure 4 shows the results from the OSATS evaluations. According to the general OSATS, 75% of the participants received a cumulative score higher than 20. The lowest score was achieved by P8 (ENT resident) and P5 (ENT specialist without experience in SS).

In all training sessions, the ChT got interrupted. The ENT specialists managed to keep its integrity until the atticotomy, whereas the residents damaged it since the preliminary dissection steps, with 2 of them transecting the nerve without notice. Assessment of the OC motility was performed appropriately by 2 participants, whereas most forgot to perform this preliminary assessment. This was scored independently as NA (not applicable) as this event was not included in the OSATS score. P5 did not manage to dissect it properly because he used the scissors instead of a hook and fractured the long process of the incus during this step.

The cutting of the stapedius received very variable scoring, as some participants did not use the scissor appropriately to cut it, and some drilled it together with the posterior crura.

The posterior crurotomy was mostly achieved after several tries, the highest score being 4 (in 3 cases). Despite fenestration receiving high scores, video recordings showed that several tries before the actual stapedotomy were performed to properly locate the drill with respect to the endoscope tip, to have both a clear view of the stapes and a stable touch of the drill on the footplate.

Crimping was performed acceptably in 4 cases, whereas 3 participants skipped this step. P6 bent the prosthesis hook inadvertently. P6 and P8 forgot to check for OC continuity at the end of the simulation.

### 3.2 Qualitative Questionnaires

**FIGURE 5:**
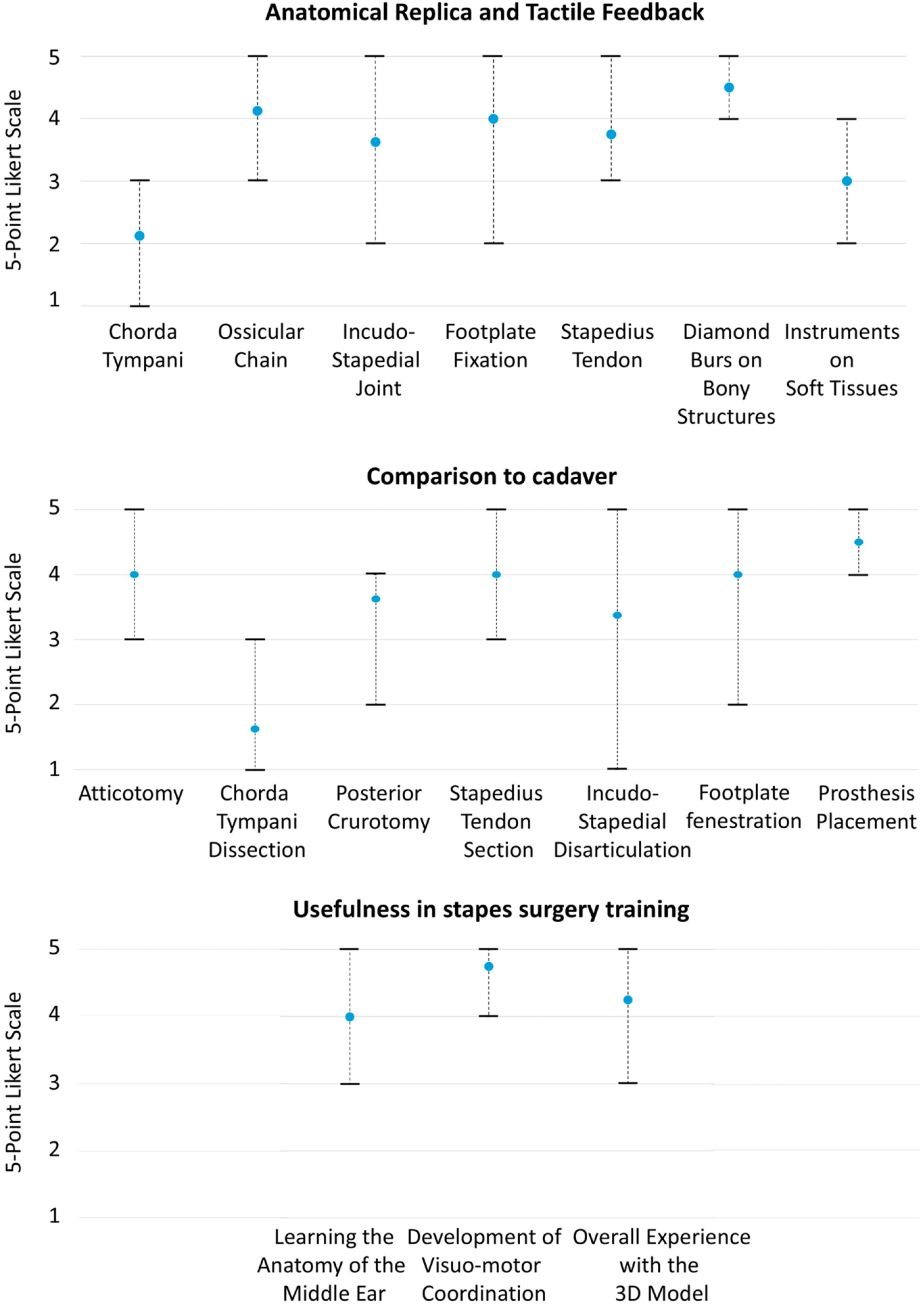
On the top of the table the results from the qualitative questionnaires regarding the realism of anatomical structure and tactile feedback of the instruments on the 3D stapes trainer, comparison between 3D stapes trainer and the cadaver in the middle and usefulness of the simulator for training purposes at the bottom. The light blue sphere represents the mean Likert‐scale value obtained from each question, while the upper and lower bars represent the maximum and minimum value, respectively.

The results of the qualitative questionnaires are reported in Figure 5. The item receiving the highest score was the tactile feedback of the burr on the bone, whereas the less appreciated part was the ChT, as confirmed by the free comments reporting the replicated nerve as too thin or too fragile. Regarding the reproducibility of the surgical steps on the 3D model, in comparison to cadaver, the items that received the highest score were stapedotomy hole creation and prosthesis placement. The 3D model was considered useful especially for developing visuomotor coordination necessary to perform the endoscopic procedure. The usefulness of the 3D model for endoscopic SS training was considered higher for the specialists than for the youngest.

## 4 Discussion

This study presents the developmental workflow and preliminary validation of the first pathological 3D printed multi‐material simulator of the ear affected by stapes fixation. This tool was specifically conceived for training in endoscopic SS, given the lack of reliable training platforms for this challenging surgery.

Previous studies have documented that clinical outcomes from SS are dependent on the surgical experience, with a volume of 50–70 procedures needed prior to obtaining stable satisfactory results [12]. Moreover, when SS is performed with a one‐hand endoscopic technique, a foundation of basic endoscopic ear surgery skills is required, and this accounts for endoscopic SS being a Level III procedure, according to the staged training program by Alicandri‐Ciufelli et al. [32, 33].

We hypothesize that the challenges in SS training may be overcome using a 3D printed simulator, which allows us to perform the procedure with an acceptable rate of replicability in a risk‐free environment. According to the feedback from the participants in this study, most of whom were naive to the endoscopic technique, our 3DST provided a valuable opportunity to practice the visuomotor coordination necessary for endoscopic surgery. Our results suggest that the training experience on the 3DST may play a role not only in exerting instrument handling and surgical gesture, but also in internalizing the procedural flow and adherence to the stepwise approach for this surgery.

The analysis of the surgical performances on the simulator confirmed the overall feasibility of the surgical steps, from freeing the ChT to assessing the OC mobility after prosthesis positioning. Seven out of 8 participants (87.5%) completed the surgery and a cumulative general OSATS score higher than 20 was obtained in 75% of cases, suggesting an acceptable performance. However, the stapes‐specific OSATS may reflect the variability of competence in the single tasks, which may be attributable to the variable experience among the subjects, as well as the possible limitations of the models in reproducing the tactile feedback of the structures. Down‐fracturing of the stapes, creating fenestration and placing the prosthesis received the highest scores, whereas assessing the chain mobility and crimping were rated as the worst. In the previously published version of the stapes‐specific OSATS, score 1 of “assessment of chain mobility” corresponded to “fracturing the ossicles”. As 5 out of 8 participants completely forgot to assess OC mobility at the beginning of surgery, we modified the score, rating this omission 1 as well.

The main innovation in the 3DST is the combination of different materials, which allows the replication of key ME structures relevant for SS, such as the ChT and the stapedial tendon, and recreates the delicate condition of a mobile malleus and incus alongside a fixed stapes. These features recreate the anatomical conditions and the tactile feedback variations encountered in patients affected by otosclerosis, which are not appreciable in any other synthetic model so far. Among the additional advantages of our model, the “multi‐parts” design facilitates easy assembly and limits the overall cost for repetitive training.

Among the 3D printed models for ear surgery available in the literature, none of them was validated for the simulation of a whole endoscopic surgical procedure. A modified Phacon TB model was developed by Nguyen et al. with an objective measurement training tool, replacing the OC with 3D printed ossicles with integrated force sensors. Measured forces during prosthesis placement and crimping were significantly lower by senior surgeons in comparison with the junior ones [25]. Other authors focused on creating 3D printed, low‐cost, and versatile trainers to enhance skills in endoscopic ear surgery, which replicated a very simplified anatomy and did not distinguish between hard and soft tissues [24, 26, 28]. Other more accurate 3D printed models, such as the one from Lähde et al., were realized from cadaveric micro‐CT (μCT) with different materials [34]. Some groups focused on the realization of simulators for teaching anatomy, such as a multi‐colored pediatric model of the ME by Jenks et al. Participants had to complete a ME anatomy test before, immediately after, and 1 week after training on the simulator with the endoscope [35]. More recently, few multi‐material models were described. In 2015, Rose et al. introduced a 3D printed TB multi‐material model to simulate the insertion of a cochlear implant [17]. Kuru et al. developed a ME model from μCT imaging, in which the ossicles were realized with selective laser melting 3D printing, while the soft tissues were cast from silicone rubber into 3D printed molds. Their model reproduced the acoustic behavior of a healthy human ME, suggesting that such a simulator may reproduce an experimental environment useful for the evaluation of prostheses [36]. In 2022, Stramiello presented a 3D printed pediatric multi‐material model including the external skin. In this study, however, the participants did not simulate any surgical procedures but validated the anatomy of the model [29]. Recently, Youner et al. evaluated the content validity of a high‐fidelity surgical ME simulator as a training tool for otolaryngology residents, with a randomized international multicenter study. The study involved 61 participants, divided into control, low‐fidelity (LF), and high‐fidelity (HF) groups. Participants in the HF group demonstrated significant improvements in both global and stapes‐specific performance scores compared to the control group. Notably, all the simulators, even the HF one, were monomaterial and consisted of the ossicles and the oval window only. Moreover, the stapedotomy technique described in the study was microscopic, representing a different scenario from the present study.

Regarding the training value of our 3DST, future studies are needed to assess its role in shortening the learning curve, as compared to traditional training methods. Although the model's utility was evident for trainees and those early in their surgical careers, its usefulness for surgeons who have already built a surgical experience in SS remains to be investigated. The results on the utility of the model from the questionnaire indicated that it was appreciated for learning the anatomy and especially for developing visual motor coordination. However, the 3DST already may hold the premises of additional educational benefits. As the endoscopic technique is the gold standard approach for SS at our Institution, the preliminary validation of the simulator was intended for this kind of approach. However, also microscopic SS could be performed on the 3D stapes trainer and further studies may evaluate its validity as a training tool for SS under the microscope. Ideally, any variant of SS technique could be simulated on the model, including Fisch's reversal steps‐stapedotomy, different approaches in opening the footplate, the use of perforators, or different types of microdrills. The possibility to simulate placement of any type of prosthesis may improve the technical skills of the surgeons and enhance their awareness of the differences in shapes and materials, boosting their confidence in tailoring the technique to the individual clinical scenarios.

There are several limitations to the model that should be mentioned. A possible drawback is the absence of the tympanomeatal flap (TMF), which prevents the performance of the critical initial step in real‐life SS. However, this step is common to all transcanal ME procedures and may be practiced in the cadaver and in the ovine model in a highly reliable way, so that the absence of the membrane allows for a quicker start in practicing the core components of SS. Participants reported that the model's ChT was prone to damage during dissection, with some trainees unintentionally transecting it. This limitation is likely due to the fragility of the synthetic material used to simulate the nerve. Particularly, Material Jetting 3D printing technology enables the printing of several polymers with varying properties, such as color and hardness, to be mixed at customizable ratios in a 3D space within the same model [37, 38, 39, 40]. However, when setting the material properties in the printing software, it is only possible to prioritize one characteristic, either hardness or color. Additionally, the production of dust from the bone replica during drilling was noted by participants as a drawback, which, while somewhat realistic, may hinder visibility.

## 5 Conclusions

The introduction of the 3DST as a multi‐material pathological model for SS training may be a significant step forward in otologic surgical education. Despite its limitations, the model provided young surgeons with a valuable platform to gain confidence in the steps of endoscopic SS, offering a high‐fidelity simulation that contributes to the development of the technical skills required in this demanding procedure. Future improvements, such as the inclusion of a TMF and refinement of the materials, may further enhance its realism and educational value.

## Conflicts of Interest

The authors declare no conflicts of interest.

## Supporting information


Data S1.

**Table S1.** General OSATS (Objective Structured Assessment of Technical Skill) Global Rating Scale, used to evaluate the performed surgical simulations.

**Table S2.** Stapedotomy‐specific OSATS (Objective Structured Assessment of Technical Skill) Rating Scale, used to evaluate the performed surgical simulations.

**Video S1.** Video showing all steps of simulation of endoscopic stapes surgery performed with the 3D printed multi‐material simulator.
