# Supplementary figures and images for: A Novel 3D Printed Multi‐Material Simulator for Endoscopic Stapes Surgery: The “3D Stapes Trainer”
Source: Laryngoscope. 2025 Apr 7;135(9):3356–63. doi: 10.1002/lary.32168 (PMC12371799; doi:10.1002/lary.32168)

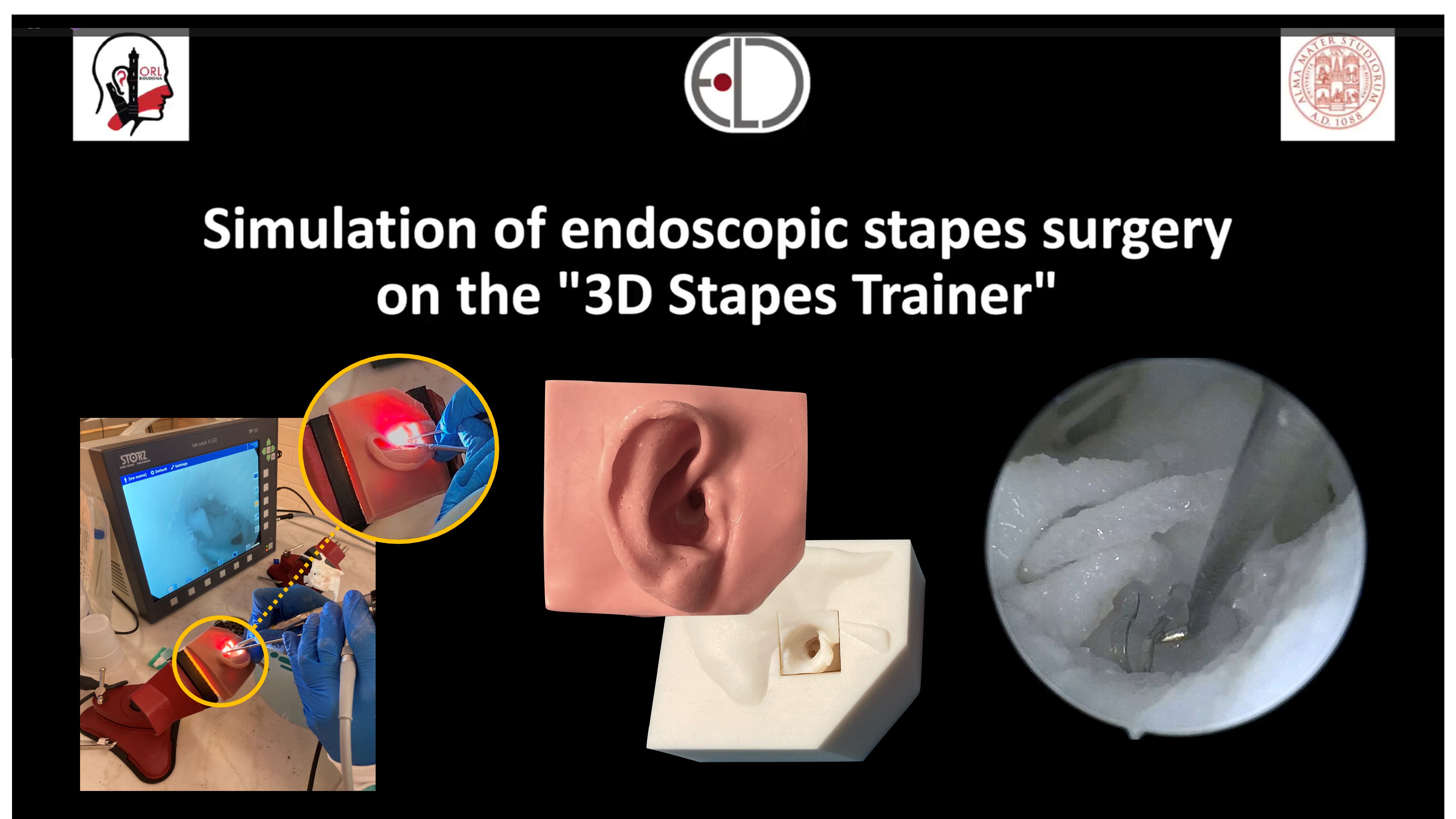

Supplement: Supplementary file 1 — Data S1. [file LARY-135-3356-s002.jpg]
