# Supplementary material for: A Novel 3D Printed Multi‐Material Simulator for Endoscopic Stapes Surgery: The “3D Stapes Trainer”
Source: Laryngoscope. 2025 Apr 7;135(9):3356–63. doi: 10.1002/lary.32168 (PMC12371799; doi:10.1002/lary.32168)
Supplement: Supplementary file 2 — Table S1. General OSATS (Objective Structured Assessment of Technical Skill) Global Rating Scale, used to evaluate the performed surgical simulations. [file LARY-135-3356-s003.docx]

**Supplementary Table S1. General OSATS Global Rating Scale**

|  | **1** | **2** | **3** | **4** | **5** |
| --- | --- | --- | --- | --- | --- |
| **Economy of movement**  **(time and motion)** | Makes unnecessary moves |  | Efficient time/motion but some unnecessary moves |  | Economy of movement and maximum efficiency |
| **Confidence of movement (instrument handling)** | Repeatedly makes tentative or awkward moves with instruments |  | Confident use of instruments although occasionally appeared stiff or awkward |  | Fluid moves with instruments and no awkwardness |
| **Respect for tissue** | Frequently used unnecessary force on tissue or caused damage by inappropriate use of instruments |  | Careful handling of tissue but occasionally caused inadvertent damage |  | Consistently handled tissues appropriately with minimal damage |
| **Flow of operation** | Imprecise, wrong technique in approaching the operative interventions |  | Careful technique with occasional errors |  | Fluent, secure and correct technique in all stages of the operative procedure |
| **Knowledge of instruments** | Frequently asks for wrong instrument or used inappropriate instrument |  | Knew names of most instruments and used appropriate instrument |  | Obviously familiar with instruments and their names |
| **Knowledge of specific procedure** | Deficient knowledge and needed instruction at most steps |  | Knew all important steps of operation |  | Demonstrated familiarity with all aspects of operation |
