# Supplementary material for: A Novel 3D Printed Multi‐Material Simulator for Endoscopic Stapes Surgery: The “3D Stapes Trainer”
Source: Laryngoscope. 2025 Apr 7;135(9):3356–63. doi: 10.1002/lary.32168 (PMC12371799; doi:10.1002/lary.32168)
Supplement: Supplementary file 3 — Table S2. Stapedotomy‐specific OSATS (Objective Structured Assessment of Technical Skill) Rating Scale, used to evaluate the performed surgical simulations. [file LARY-135-3356-s004.docx]

**Supplementary Table S2: Stapedotomy-specific OSATS Rating Scale**

|  | **1** | **2** | **3** | **4** | **5** |
| --- | --- | --- | --- | --- | --- |
| **1.Free chorda tympani** | Transected the nerve without notice |  | Handles the nerve carefully |  | Meticulously dissected nerve and moved it |
| **2. Assess ossicular mobility** | Forgot to assess or fractured ossicles |  | Gently palpated but not able to assess mobility |  | Carefully palpated and demonstrated stapes fixation |
| **3. Transect incudo-stapedial joint** | Dislocated incus or stapes |  | Transected after several tries |  | Smooth perfect transection |
| **4. Cut stapedius** | Unable to cut or damaged stapes |  | Transected after several tries |  | Smooth perfect transection |
| **5. Cut posterior crura of stapes** | Fractured stapes or unable to cut |  | Transected after several tries |  | Smooth perfect transection |
| **6. Down-fracture**  **stapes** | Up-fractured or hit facial nerve |  | Down-fractured after several tries |  | Smooth perfect down fracture |
| **7. Create fenestration** | Unable or went too deep into vestibule |  | Careful but unable to make correct dimension or too much pressure on incus |  | Smooth perfect fenestration with correct dimensions |
| **8. Place prosthesis** | Dropped and bent prosthesis |  | Able to place over incus after several tries |  | Smooth handling and placement |
| **9. Crimp prosthesis** | Fractured incus or bent prosthesis |  | Able to crimp after several tries |  | Smooth crimping with adequate tightness |
| **10. Check for ossicular continuity** | Forgot or placed too much pressure |  | Checked for mobility but not very smooth |  | Checked for mobility and depth of prosthesis in vestibule |
